# Black Men’s Health and Aging Across the Life Course

**DOI:** 10.1093/geroni/igaf122.1300

**Published:** 2025-12-31

**Authors:** Roland Thorpe, Carl Hill

## Abstract

Much of the research on Black men tend not to focus on concepts that have been found to be important determinants of health for Black men. Nor, do they seek to identify sources of resilience that may be protective of health. This symposium contains a collection of papers that discuss some key social determinants of health (SDOH) that can provide insights to advance our understanding of Black men’s health and aging. Esiaka investigated whether active coping moderates the association between neighborhood segregation and Subjective cognitive decline (SCD) in Black American men. Findings indicate the association between neighborhood segregation and SCD depends on active coping levels. Bruce and colleagues examined how heterogeneity of income influences correlates of cognitive impairment among Black men using data from the 2016 Health and Retirement Study. These investigators found that age and poor health were significant for men reporting incomes below the national median while post-secondary education and depressive symptoms were associated with any cognitive impairment among Black men with incomes over $50,000. Jones examined equity-focused approaches to preserve brain health in aging Black men with HIV to reduce disparities in cognitive and cardiovascular outcomes. His work highlighted the importance of the need for an interdisciplinary team to advance the understanding of the link between HIV, cardiovascular outcomes and cognitive outcomes. These presentations will bolster our knowledge on SDOH among Black men across the life course.

